# Incidence of neutropenia in patients with ticlopidine/Ginkgo biloba extract combination drug for vascular events: A post-marketing cohort study

**DOI:** 10.1371/journal.pone.0217723

**Published:** 2019-06-05

**Authors:** Han-Gil Jeong, Jae Sun Yoon, Juneyoung Lee, Hee-Joon Bae

**Affiliations:** 1 Department of Neurology and Cerebrovascular Center, Seoul National University Bundang Hospital, Seongnam, Republic of Korea; 2 Department of Biostatistics, Korea University, Seoul, Korea; National Institute of Technology Calicut, INDIA

## Abstract

**Background and purpose:**

The ticlopidine/Ginkgo biloba ext. combination drug (Yuclid) is used as an antiplatelet agent for prevention of vascular events since its approval in 2008. The purpose of this study is to explore the safety of ticlopidine/Ginkgo biloba combination, mainly regarding the incidence of neutropenia, through a post-marketing surveillance study.

**Methods:**

From March 2009 to October 2015, a total of 4839 subjects had been enrolled in this study. The enrollments were conducted by 152 doctors of 89 hospitals according to the regulations for post-marketing surveillance programs in Korea. If a subject was administered the drug once, he/she was included in the safety analysis set for any adverse events and bleedings, and the primary safety evaluation regarding neutropenia was conducted in subjects who completed 3-month blood test follow-up. We predefined that 1% reduction in neutropenia incidence by ticlopidine/Ginkgo biloba ext. combination from the previously reported incidence of ticlopidine of 2.3% was clinically meaningful.

**Results:**

Among the safety analysis set of 4831 patients (99.8% of the enrolled subjects), 3150 (65.1%) completed evaluation for neutropenia at 3 months which is the primary safety endpoint. The major causes of dropout were no follow-up visit at 3 months (n = 1016) and violation of the follow-up period (n = 503). Nine patients experienced neutropenia (Absolute neutrophil count [ANC] ≤ 1200mm^3^) and the estimated cumulative incidence at 3 months is 0.29% (95% confidence interval, 0.13%– 0.54%). Severe neutropenia (ANC ≤ 450mm^3^) did not occur in any patients.

**Conclusions:**

The incidence of neutropenia with addition of Ginkgo biloba ext. to ticlopidine may be lower than the previously reported incidence of neutropenia with ticlopidine, which needs to be confirmed in randomized controlled trials.

## Introduction

Ticlopidine, an antiplatelet drug in the thienopyridine family, demonstrated its efficacy for secondary prevention of stroke in patients with previous cerebral ischemia in two large clinical trials, the Canadian American Ticlopidine Study (CATS) and Ticlopidine Aspirin Stroke Study (TASS).[1, 2] Moreover, the combination of ticlopidine and aspirin was effective in reducing stent thrombosis and vascular events after coronary stent placement.[3, 4] From these successful trials, ticlopidine became the most commonly used antiplatelet agent next to aspirin in Europe and the United States.[5]

After widespread use of ticlopidine, hematologic abnormalities including neutropenia associated with ticlopidine had been reported. Since the incidence of neutropenia was about 2% and fully reversible after stopping ticlopidine, blood test monitoring was recommended during the first 3 months after starting ticlopidine.[6] However, a succeeding thienopyridine-based antiplatelet drug, clopidogrel, demonstrated a better safety for neutropenia than ticlopidine.[7–10] And clopidogrel has largely replaced ticlopidine in clinical practice.

In the course of time, it has been reported that persons with clopidogrel unresponsiveness related to a CYP2C19 reduced-function allele are at an increased risk of cardiovascular events when treated with clopidogrel and the prevalence of this clopidogrel unresponsiveness is not uncommon, especially in Asians.[11, 12] In this case, ticlopidine can be an alternative, if neutropenia is avoidable to some degree.[13, 14] The suggested cause of neutropenia is a toxic and reactive metabolite of ticlopidine which is converted in activated neutrophil and its precursors.[15] Gingko biloba extract, a reactive oxygen species scavenger, showed the potential to reduce neutropenia when administered concomitantly with ticlopidine in a preclinical study.[16]

A combination drug of ticlopidine/Ginkgo biloba ext. (Yuclid) was developed since the combination demonstrated an enhanced antithrombotic effect in both experimental and clinical studies.[17–20] Ticlopidine/Ginkgo biloba ext. has been used as an anti-platelet agent for prevention of vascular events since it was approved in Korea in 2008. The purpose of this study is to investigate the safety of the ticlopidine/Ginkgo biloba ext combination in terms of neutropenia through a large prospective post-marketing surveillance study in Korea.

## Methods

### Study population and sample size estimation

From March 2009 to October 2015, we conducted a post-marketing surveillance study on consecutive patients prescribed with a combination of ticlopidine/Ginkgo biloba ext. (250/80mg) from 152 doctors in 89 institutions. This post-marketing surveillance study was approved by Ministry of Food and Drug Safety, Republic of Korea and the Institutional Review Board of the following participating hospitals: Ajou University Hospital, Bumin Hospital Busan, Bundang Jesaeng Hospital, Catholic Kwandong University International St. Mary’s Hospital, Changwon Fatima Hospital, Cheonan Chungmu Hospital, Chonbuk National University Hospital, Chonnam National University Hospital, Chosun University Hospital, Chungbuk National University Hospital, Chungnam National University Hospital, Daedong Hospital, Daegu Catholic Univ. Medical Center, Daegu Fatima Hospital, Daejeon Medical Center Eulji University, Daejeon Sun Hospital, Dankook University Hospital, Donghea Dong In Hospital, Eulji Medical Center Eulji University, Gachon University Gil Medical Center, Gangnam Severance Hospital, Gangneung Asan Hospital, Gyeongsang National University Hospital, Hanyang University Medical Center, Inha University Hospital, Inje University Sanggye Paik Hospital, Inje University Sanggye Paik Hospital, Jung Ang Hospital, Kangbuk Samsung Hospital, Kangwon National University Hospital, Keimyung University Dongsan Medical Center, Konkuk University Medical Center, Konyang University Hospital, Korea University Anam Hospital, Korea University Guro Hospital, Kwangju Christian Hospital, Kyunghee University Medical Center, Mokpo Hankook Hospital, Myongji Hospital, National Health Insurance Service Ilsan hospital, Pusan National University Yangsan Hospital, Samsung Changwon Hospital, Seoul National University Bundang Hospital, Seoul National University Hospital, Wonkwang Sanbon Hospital, Yeungnam University Medical Center, and Pyeongtaek International Hospital. Written informed consent was obtained from the participants.

The null hypothesis was the combination of ticlopidine/Ginkgo biloba ext. had the same incidence of neutropenia as ticlopidine alone, which was assumed to be 2.3% based on the results of the TASS study[2]. Under the alternative hypothesis, the neutropenia incidence of ticlopidine/Ginkgo biloba was assumed to be 1.3%. With the superiority margin of 1.8%, a sample size of 3,880 patients would provide a power of 81% by a binomial test under one-tailed significance level of 5%. This sample size could estimate the anticipated neutropenia incidence of 2.3% with 95% confidence interval of 1.8% to 2.3% under the null hypothesis, and 1.3% with 95% confidence interval of 0.95% to 1.65% under the alternative hypothesis. Considering a dropout rate of 20%, the final sample size was determined to be 4,850. Entry requirements included transient ischemic attack/ischemic stroke, chronic peripheral arterial occlusion or coronary artery disease. Patients with the following conditions were excluded: age less than 40, a history of peptic ulcer or upper gastrointestinal bleeding, a history of life-threatening disease including cancer, a history of hypersensitivity reaction to ticlopidine or Ginkgo biloba ext., or those already taking ticlopidine. Information on demographics, comorbidities including hepatic and renal disease, allergy, medications, alcohol consumption, and smoking were collected.

### Outcome assessments

The primary objective of this study was to examine the safety of ticlopidine/Ginkgo biloba ext. by estimating the incidence of neutropenia at 3 months after starting the study drug. The secondary objective was to investigate the incidence of bleeding and other adverse events. Neutropenia is defined as an absolute neutrophil count (ANC) of less than 1200 per mm^3^ and severe neutropenia is defined as an ANC of less than 450 per mm^3^. Bleeding events were defined and categorized into severe, moderate, and minor bleedings using the Global Use of Strategies to Open Occluded Arteries (GUSTO) definitions.[21] Vascular events were defined as the occurrence of stroke (ischemic or hemorrhagic), myocardial infarction, or cardiovascular death. The primary safety population were those who completed a 3-month laboratory test to measure ANC. The secondary safety population was those who were evaluated for bleeding events and other adverse events.

### Statistical analysis

We calculated the cumulative incidence of neutropenia at 3 months after taking ticlopidine/gingko biloba ext. and its 95% CI. The difference in neutropenia incidence by age, sex, and treatment duration was assessed by Fisher's exact or chi-square tests. In the secondary safety population, the incidence of bleeding events and other adverse events was calculated. Sample size was calculated using PASS 2005 software. Statistical analyses were performed using SAS software version 8.2 (SAS Institute, Cary, NC, USA) and R version 3.5.1 (R Development Core Team, Vienna, Austria). Two-sided P values of < 0.05 were considered statistically significant.

### Sensitivity analysis

Based on a meta-analysis of four clinical trials evaluating the long-term use of ticlopidine, a neutropenia incidence of ticlopidine at 3 months was estimated using a mixed-effect Poisson regression model.[22] As a sensitivity analysis, 1) we added patients who were excluded because their follow-up ANC tests were out of the predetermined window period (n = 503), and 2) we imputed the neutropenia events in the study subjects, who were excluded from the primary safety analysis due to no follow-up ANC test (n = 1178), using the point estimate of neutropenia in the above meta-analysis, and calculated a cumulative incidence of neutropenia in the entire population by adding these patients too.

## Results

A total of 4839 cases were collected, of which 4831 (99.8%) met the inclusion/ exclusion criteria. Among these 4831 patients, 696 (14.4%) discontinued treatment prematurely during the 3-month follow-up period. Major reasons for discontinuation were follow-up loss (n = 421, 60.5%) and adverse events (n = 119, 17.1%) (S1 Table). The primary safety evaluation was conducted in 3150 (65.2%) cases after excluding 1681 (34.9%), who violated the prespecified criteria of follow-up assessment (Table 1). The secondary safety evaluation was conducted in all the eligible cases (n = 4831) (S1 Fig). Men were 52% and the mean age was 67.0 ± 10.6 year. The major indication of ticlopidine/Ginkgo biloba ext. was transient ischemic attack or ischemic stroke (82.9%). Most patients were treated in outpatient clinic settings (74.3%). A daily dose of ticlopidine was 500 mg in 77.5% and 250 mg in 22.5% of patients. The number of patients taking the study drug over 90 days was 2,739 (67.5%) (S2 Table). Mean baseline ANC of these patients was 4160 ± 1690 per mm^3^.

**Table 1:** Baseline characteristics.

	Total (N = 4831)	Not eligible for primary safety evaluation (N = 1681)	Primary safety population (N = 3150)	*p*-value
Sex				<0.01
Male	2513 (52.0%)	816 (48.5%)	1697 (53.9%)	
Female	2318 (48.0%)	865 (51.5%)	1453 (46.1%)	
Age	67.0±10.6	67.7±10.8	66.7±10.5	<0.01
Indication				
Peripheral artery disease	196 (4.1%)	80 (4.8%)	116 (3.7%)	0.08
TIA/Stroke	4017 (83.2%)	1409 (83.8%)	2608 (82.8%)	0.39
Coronary artery disease	681 (14.1%)	217 (12.9%)	464 (14.7%)	0.09
Physician Specialty				<0.01
Thoracic surgery	80 (1.7%)	12 (0.7%)	68 (2.2%)	
Cardiology	444 (9.19%)	89 (5.29%)	355 (11.3%)	
Endocrinology	144 (3.0%)	104 (6.2%)	40 (1.3%)	
Internal medicine	150 (3.1%)	30 (1.8%)	120 (3.8%)	
Neurology	3679 (76.2%)	1355 (80.6%)	2324 (73.8%)	
Neurosurgery	322 (6.7%)	80 (4.8%)	242 (7.7%)	
Rehabilitation	12 (0.3%)	11 (0.7%)	1 (0.03%)	
Care setting*				0.02
Outpatient	3591 (74.3%)	1259 (74.9%)	2332 (74.0%)	
Inpatient	562 (11.6%)	214 (12.7%)	348 (11.0%)	
Both	677 (14.0%)	207 (12.3%)	470 (14.9%)	
Alcohol consumption†	1046 (21.7%)	349 (20.8%)	697 (22.1%)	0.30
Smoking†				<0.01
Non-smoker	3376 (70.0%)	1248 (74.4%)	2128 (67.6%)	
Current smoker	847 (17.6%)	268 (16.0%)	579 (18.4%)	
Ex-smoker	603 (12.5%)	162 (9.65%)	441 (14.0%)	
Daily drug dose‡				<0.01
250mg/d	1077 (22.5%)	323 (19.5%)	754 (24.0%)	
500mg/d	3717 (77.5%)	1329 (80.4%)	2387 (75.9%)	

Values are mean±standard deviation or number (%).

*4 missing data.

†5 missing data.

‡35 missing data, 125mg/d (n = 1) and 750mg/d (n = 1), presented with ticlopidine dose.

TIA = Transient ischemic attack

The incidence of neutropenia, the primary safety outcome, was evaluated based on 3,150 patients (65.1%) who had the follow-up ANC test and completed the follow-up at the prespecified time point (from 3 months minus 2 weeks to 3 months plus 2 months). Nine patients developed neutropenia (the case details are presented in S3 Table) and the estimated incidence was 0.29% (95% CI; 0.13% to 0.54%). The severity of neutropenia was mild to moderate (450/mm^3^ < ANC ≤ 1200/mm^3^) in 9 patients and there was no case with severe neutropenia (ANC ≤ 450/mm^3^) (Table 2). All episodes resolved after ticlopidine/Ginkgo biloba ext. was discontinued and there was no further related adverse event. The incidence of neutropenia was not different by sex, age groups and duration of treatment (Table 3).

**Table 2:** Incidence of neutropenia and bleeding events.

	n (%)	95% CI
**Neutropenia**		
No (ANC > 1200 mm^3^)	3141 (99.71)	(99.46, 99.87)
Yes (ANC ≤ 1200 mm^3^)	9 (0.29)	(0.13, 0.54)
Mild to moderate (450 mm^3^ < ANC ≤ 1200mm^3^)	9 (0.29)	(0.13, 0.54)
Severe (ANC ≤ 450mm^3^)	0 (0.00)	-
**Bleeding events**		
No	4806 (99.48)	(99.24, 99.66)
Yes	25 (0.52)	(0.34, 0.76)
Severe†	3 (12.00)	(2.55, 31.22)
Moderate†	3 (12.00)	(2.55, 31.22)
Mild†	19 (76.00)	(54.87, 90.64)

ANC = absolute neutrophil count, CI = confidence interval

**Table 3:** Incidence of neutropenia by sex, age group and treatment duration <primary safety population>.

		Neutropenia		
Subgroups	Cases	N (%)	95% CI	P-value
Sex				0.52
Male	1697	6 (0.35)	(0.13, 0.77)	
Female	1453	3 (0.21)	(0.04, 0.60)	
Age group				0.26
40–49	209	1 (0.48)	(0.01, 2.64)	
50–59	605	1 (0.17)	(0.00, 0.92)	
60–69	938	5 (0.53)	(0.17, 1.24)	
70–79	1089	1 (0.09)	(0.00, 0.51)	
80 or more	309	1 (0.32)	(0.01, 1.79)	
Duration				1.00
≤ 5 days	1	0 (0)	-	
≥ 6 days, < 2 weeks	1	0 (0)	-	
≥ 2 weeks, < 30 days	2	0 (0)	-	
≥ 30 days, < 60 days	8	0 (0)	-	
≥ 60 days, < 90 days	696	2 (0.29)	(0.03, 1.03)	
≥ 90 days	2052	7 (0.34)	(0.14, 0.70)	
Missing	390			

The bleeding event occurred in 25 patients (the estimated incidence, 0.52%; 95% CI, 0.34% to 0.76%). The severity of bleeding events was mild in 19 patients (76%), moderate in 3 (12%), and severe in 3 (12%) (Table 2). The incidence of bleeding events was higher in female than male (male, 0.32% [95% CI, 0.14% to 0.63%]; female, 0.73% [0.43% to 1.17%]; p-value, 0.045), but the majority was minor bleedings. The incidence of bleeding was highest in those with the treatment duration of 30 to 59 days but was not different by age groups (S4 Table). A total of 425 adverse events (AE) occurred in 349 patients (7.22%); 42 serious AEs in 38 patients (0.79%). The most frequent AE was dizziness (n = 29, 0.60%), followed by pruritus (n = 25, 0.52%), rash (n = 21, 0.43%), dyspepsia (n = 19, 0.39%), and nausea (n = 18, 0.37%). Regarding vascular events, stroke occurred in 5 patients (4 ischemic stroke and 1 undetermined) and no myocardial infarction or cardiovascular death occurred during the follow-up period.

**Fig 1:**
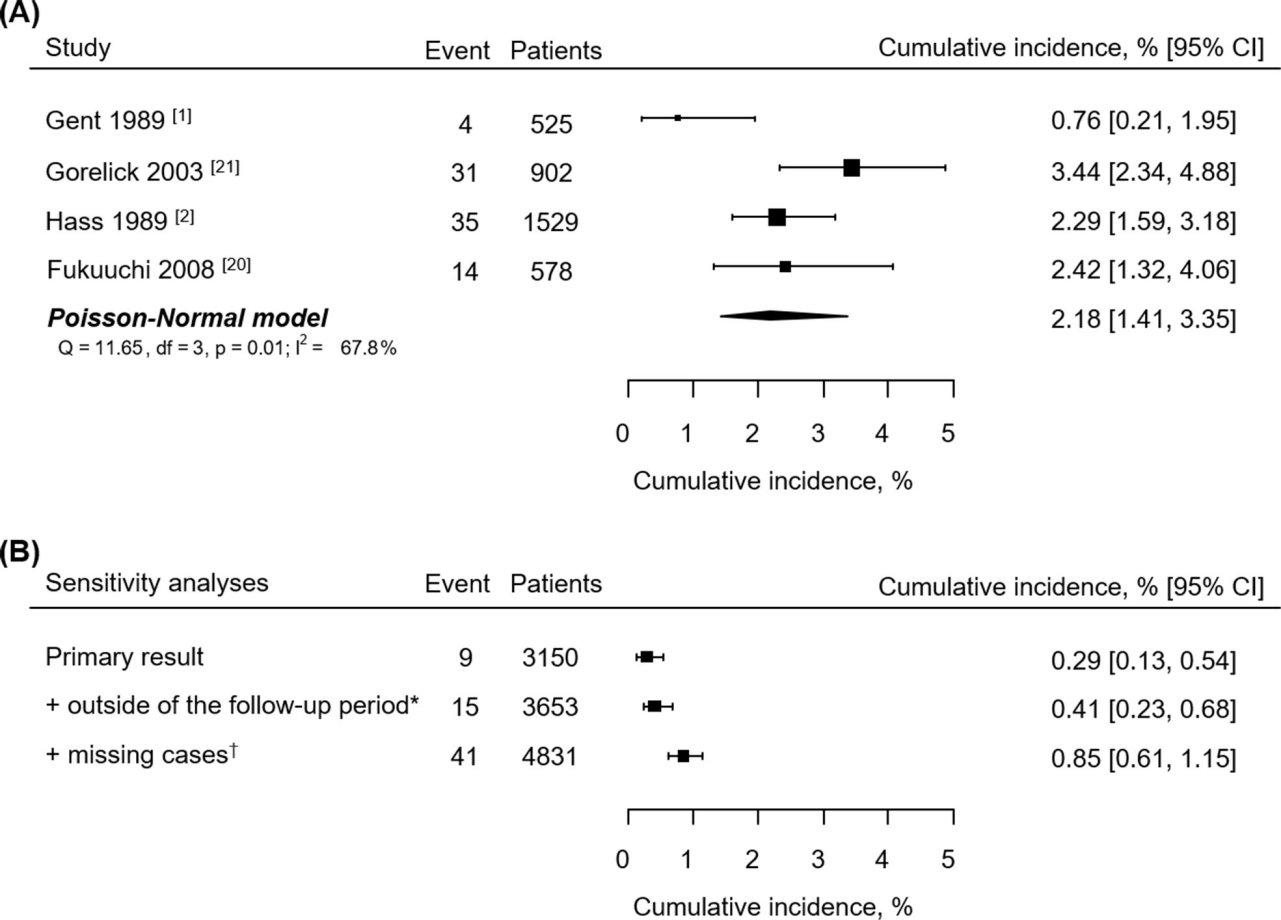
**(A) meta-analysis of cumulative incidence of neutropenia by ticlopidine use and (B) sensitivity analyses of our results.** * Inclusion of the cases with the follow-up ANC test outside of the window period (n = 503). † Incidence of neutropenia in the cases with no follow-up ANC test (n = 1178) is assumed to be same with the result of a meta-analysis.

We performed post hoc sensitivity analyses to examine the robustness of the study results despite its high drop-out rate. A meta-analysis of the previous 4 ticlopidine trials showed that the 3-month cumulative incidence of neutropenia was 2.19% [1.45% to 3.30%] (Fig 1A). When adding the cases with the follow-up ANC test outside of the window period, the incidence of neutropenia incidence was 0.41% [0.23% to 0.68%]. When the incidence of neutropenia in the excluded patients due to no follow-up ANC test is assumed to be same with the result of a meta-analysis, the neutropenia incidence at 3 months in the ticlopidine/Ginkgo biloba ext. group was 0.85% [0.61% to 1.15%] (Fig 1B). The neutropenia incidences calculated with different ANC cut-offs and the comparison to the previous studies are provided in S6 Table.

## Discussions

We examined the incidence of neutropenia in patients who received the combination of ticlopidine/Ginkgo biloba ext. through a post-marketing surveillance study. The majority of patients were prescribed the study drug for secondary prevention of transient ischemic attack or stroke. In the study subjects who taking the combination of ticlopidine/Ginkgo biloba ext., the incidence of neutropenia was 0.29% which was lower than the previous reports for ticlopidine alone. There was no case of severe neutropenia in our study.

The incidence of neutropenia in patients taking ticlopidine was about 2.2% in the meta-analysis of the previous 4 trials; the trial details are summarized in S5 Table.[1, 2, 23, 24] These studies have some degree of heterogeneity regarding neutropenia incidence, largely because of differences in the definition of neutropenia among studies. The definition of neutropenia in our study (ANC < 1200/mm^3^) was the same as the TASS trial.[2] While CATS[1] and TASS trial[2] included more than 70% of Caucasian, Gorelick et al.[24] and Fukuuchi et al.[23] studied only African American and Japanese, respectively, but the occurrence of neutropenia in patients taking ticlopidine does not seem to differ by race. Like the previous studies, the risk factor for neutropenia was not determined and the incidence of neutropenia did not differ by age, sex and duration of treatment in our study.

The dropout rate for the primary safety evaluation (neutropenia) was quite high. The major cause of dropout was no follow-up visit at 3 months (n = 1016, 60.4%) or violation of the follow-up period (n = 503, 29.9%). Premature discontinuation rate was not high compared to the previous studies. The baseline characteristics were slightly different between the excluded cases and the primary safety population, but there has been no known risk factor for to neutropenia upto now. However, the possible underestimation due to unselective follow-up loss or healthy people bias is justifying post-hoc sensitivity analyses. The neutropenia incidence of the dropped-out patients was replaced with a point estimate of the meta-analysis and the neutropenia incidence of the whole population was calculated. The point estimates of neutropenia incidence in the primary safety population and the sensitivity analysis populations did not exceed 1.3%, which was predetermined for sample size calculation, and even the upper margins of their 95% CIs did not too, all of which are supporting the robustness of the results of this study.

Ticlopidine may still be potentially useful in certain cases if the neutropenia could be avoidable. The subgroup studies of the TASS[2] suggested more beneficial effects of ticlopidine in non-whites or minor stroke.[25, 26] Ticlopidine has not been thoroughly studied for efficacy and safety for Asians who have higher resistance to clopidogrel than westerners. In the substudy of the Clopidogrel in High-Risk Patients with Acute Non-disabling Cerebrovascular Events (CHANCE) trial, the combination of clopidogrel and aspirin did not reduce the risk of a new stroke in the carriers of the CYP2C19 loss-of-function alleles which are especially prevalent in East Asians.[12, 27] Since the antiplatelet effect of ticlopidine is known to be unaffected by the loss of function of CYP2C19,[14] ticlopidine needs to be reevaluated regarding the usefulness for patients with clopidogrel resistance in this era of precision medicine.

**Fig 2:**
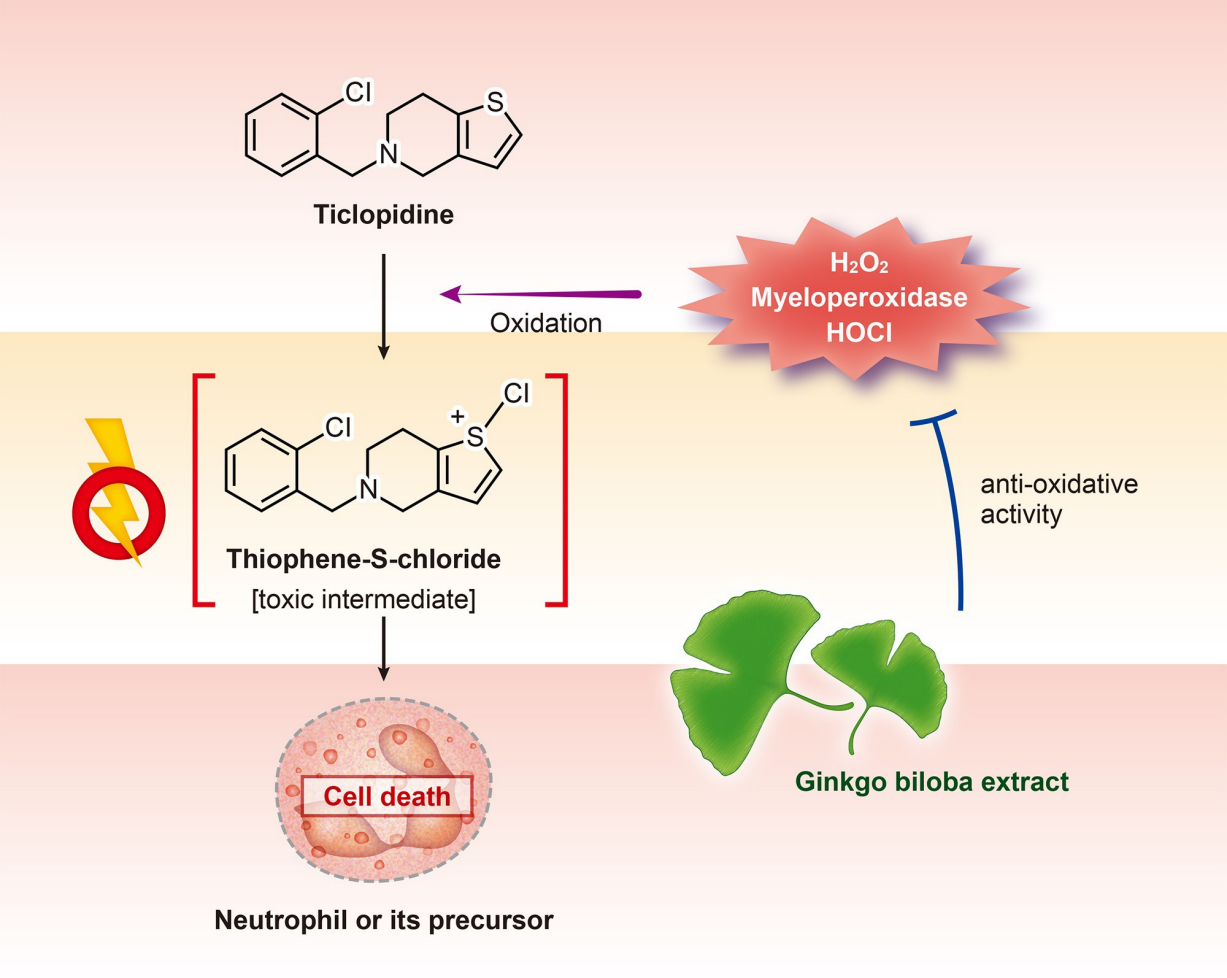
Schematic representation of protective effects of Ginkgo biloba ext. for neutropenia induced by ticlopidine.

The potential mechanism of ticlopidine-induced neutropenia is a toxicity from a reactive intermediate, Thiophene-S-chloride, formed by oxidation due to hydrogen peroxide or myeloperoxidase in activated neutrophils or neutrophil precursors (Fig 2).[15] The anti-oxidative effect of Ginkgo biloba ext. gained attention due to its protective effect for neuronal death from oxidative stress in the brain.[28] Ginkgo biloba ext. has also been reported to reduce cellular toxicity by reactive oxygen species in various immune cells.[29–32] In a preclinical study, the combination of ticlopidine and Ginkgo biloba ext. reduced the bone marrow toxicity and the neutropenia induced by ticlopidine,[16] which may be associated with the findings of the current study.

A few things should be clarified for the interpretation of this study. The drop-out rate of patients in this study was quite high, which was inevitable in a post-marketing surveillance study.[33] Due to the structural problems of the Korean healthcare system, Koreans have a low utilization rate of a usual source of medical care and doctor shopping is quite common, which may have caused frequent follow-up losses during the surveillance.[34] The high drop-out rate could cause healthy people bias, risk factor imbalance, unselective follow-up loss related to neutropenia occurrence, and finally underestimation of the neutropenia incidence in this study. However, the sensitivity analyses that conservatively estimated the neutropenia incidence in excluded cases did not significantly alter the results. This study should be considered as just generating a hypothesis, and a randomized controlled trial is required for confirming the results. The results of blood tests were collected twice (baseline and 3 months), which was less frequent than other studies, may also have caused underestimation.

In this post-marketing surveillance-based study, ticlopidine/Ginkgo biloba ext. showed a tolerable range of neutropenia incidence which is lower than expected in ticlopidine alone. If the enhanced safety of ticlopidine/Ginkgo biloba ext. is confirmed, ticlopidine/Ginkgo biloba ext. can be used as an alternative for patients with clopidogrel resistance, especially for Asians.

## Supporting information

S1 Fig Disposition of study subjects. (TIF)

S1 Table Medication status of study drug. (PDF)

S2 Table Trial drug information. (PDF)

S3 Table Details of subject information with neutropenia at 3 months. (PDF)

S4 Table Bleeding events by sex, age group and treatment duration. (PDF)

S5 Table Characteristics of the included studies for meta-analysis. (PDF)

S6 Table Neutropenia incidence defined with different cut-offs of absolute neutrophil count and comparison to the previous studies. (PDF)

## References

[1] Gent M, Easton JD, Hachinski V, Panak E, Sicurella J, Blakely J, et al The Canadian American ticlopidine study (CATS) in thromboembolic stroke. The Lancet. 1989;333(8649):1215–20. 10.1016/s0140-6736(89)92327-1 2566778

[2] Hass WK, Easton JD, Adams HP Jr, Pryse-Phillips W, Molony BA, Anderson S, et al A randomized trial comparing ticlopidine hydrochloride with aspirin for the prevention of stroke in high-risk patients. New England Journal of Medicine. 1989;321(8):501–7. 10.1056/NEJM198908243210804 2761587

[3] Leon MB, Baim DS, Popma JJ, Gordon PC, Cutlip DE, Ho KK, et al A clinical trial comparing three antithrombotic-drug regimens after coronary-artery stenting. New England Journal of Medicine. 1998;339(23):1665–71. 10.1056/NEJM199812033392303 9834303

[4] Schömig A, Neumann F-J, Kastrati A, Schühlen H, Blasini R, Hadamitzky M, et al A randomized comparison of antiplatelet and anticoagulant therapy after the placement of coronary-artery stents. New England Journal of Medicine. 1996;334(17):1084–9. 10.1056/NEJM199604253341702 8598866

[5] Steinhubl SR, Tan WA, Foody JM, Topol EJ, Investigators E. Incidence and clinical course of thrombotic thrombocytopenic purpura due to ticlopidine following coronary stenting. Jama. 1999;281(9):806–10. 10071001 10.1001/jama.281.9.806

[6] Roche. Ticlid (ticlopidine): US Food & Drug Administration; 2001.

[7] Bertrand ME, Rupprecht H-Jr, Urban P, Gershlick AH. Double-blind study of the safety of clopidogrel with and without a loading dose in combination with aspirin compared with ticlopidine in combination with aspirin after coronary stenting: the clopidogrel aspirin stent international cooperative study (CLASSICS). Circulation. 2000;102(6):624–9. 10931801 10.1161/01.cir.102.6.624

[8] Taniuchi M, Kurz HI, Lasala JM. Randomized comparison of ticlopidine and clopidogrel after intracoronary stent implantation in a broad patient population. Circulation. 2001;104(5):539–43. 11479250 10.1161/hc3001.093435

[9] Müller C, Büttner HJ, Petersen J, Roskamm H. A randomized comparison of clopidogrel and aspirin versus ticlopidine and aspirin after the placement of coronary-artery stents. Circulation. 2000;101(6):590–3. 10673248 10.1161/01.cir.101.6.590

[10] Committee CS. A randomised, blinded, trial of clopidogrel versus aspirin in patients at risk of ischaemic events (CAPRIE). The Lancet. 1996;348(9038):1329–39. 10.1016/s0140-6736(96)09457-3 8918275

[11] Mega JL, Close SL, Wiviott SD, Shen L, Hockett RD, Brandt JT, et al Cytochrome p-450 polymorphisms and response to clopidogrel. New England Journal of Medicine. 2009;360(4):354–62. 10.1056/NEJMoa0809171 19106084

[12] Wang Y, Zhao X, Lin J, Li H, Johnston SC, Lin Y, et al Association between CYP2C19 loss-of-function allele status and efficacy of clopidogrel for risk reduction among patients with minor stroke or transient ischemic attack. Jama. 2016;316(1):70–8. 10.1001/jama.2016.8662 27348249

[13] Aleil B, Rochoux G, Monassier JP, Cazenave JP, Gachet C. Ticlopidine could be an alternative therapy in the case of pharmacological resistance to clopidogrel: a report of three cases. Journal of Thrombosis and Haemostasis. 2007;5(4):879–81. 10.1111/j.1538-7836.2007.02338.x 17403206

[14] Maeda A, Ando H, Asai T, Ishiguro H, Umemoto N, Ohta M, et al Differential impacts of CYP2C19 gene polymorphisms on the antiplatelet effects of clopidogrel and ticlopidine. Clinical Pharmacology & Therapeutics. 2011;89(2):229–33. 21178986 10.1038/clpt.2010.268

[15] Liu ZC, Uetrecht JP. Metabolism of ticlopidine by activated neutrophils: implications for ticlopidine-induced agranulocytosis. Drug metabolism and disposition. 2000;28(7):726–30. 10859143

[16] Ahn HS. Inhibitory Effects of Ginkgo biloba extract (EGb 761) on Ticlopidine-induced Neutropenia. Dongduk Pharm Res. 2007;11:1–11.

[17] Kim B-H, Kim K-P, Lim KS, Kim J-R, Yoon SH, Cho J-Y, et al Influence of Ginkgo biloba extract on the pharmacodynamic effects and pharmacokinetic properties of ticlopidine: an open-label, randomized, two-period, two-treatment, two-sequence, single-dose crossover study in healthy Korean male volunteers. Clinical therapeutics. 2010;32(2):380–90. 10.1016/j.clinthera.2010.01.027 20206795

[18] Hong JM, Shin DH, Lim YA, Lee JS, Joo IS. Ticlopidine with Ginkgo Biloba extract: a feasible combination for patients with acute cerebral ischemia. Thrombosis research. 2013;131(4):e147–e53. 10.1016/j.thromres.2013.01.026 23477707

[19] Kim YS, Pyo MK, Park KM, Park PH, Hahn BS, Wu SJ, et al Antiplatelet and antithrombotic effects of a combination of ticlopidine and ginkgo biloba ext (EGb 761). Thrombosis research. 1998;91(1):33–8. 9700851 10.1016/s0049-3848(98)00075-9

[20] Chung J-W, Kim SJ, Hwang J, Lee MJ, Lee J, Lee K-Y, et al Comparison of clopidogrel and ticlopidine/Ginkgo biloba in combination with aspirin in patients with clopidogrel resistance and carotid stenting. Frontiers in neurology. 2019;10:44 10.3389/fneur.2019.00044 30761076 PMC6363652

[21] Investigators GUSTO. An international randomized trial comparing four thrombolytic strategies for acute myocardial infarction. New England Journal of Medicine. 1993;329(10):673–82. 10.1056/NEJM199309023291001 8204123

[22] Stijnen T, Hamza TH, Özdemir P. Random effects meta‐analysis of event outcome in the framework of the generalized linear mixed model with applications in sparse data. Statistics in medicine. 2010;29(29):3046–67. 10.1002/sim.4040 20827667

[23] Fukuuchi Y, Tohgi H, Okudera T, Ikeda Y, Miyanaga Y, Uchiyama S, et al A randomized, double-blind study comparing the safety and efficacy of clopidogrel versus ticlopidine in Japanese patients with noncardioembolic cerebral infarction. Cerebrovascular diseases. 2008;25(1–2):40–9. 10.1159/000111498 18033957

[24] Gorelick PB, Richardson D, Kelly M, Ruland S, Hung E, Harris Y, et al Aspirin and ticlopidine for prevention of recurrent stroke in black patients: a randomized trial. Jama. 2003;289(22):2947–57. 10.1001/jama.289.22.2947 12799402

[25] Weisberg LA, Group TASS. The efficacy and safety of ticlopidine and aspirin in non‐whites Analysis of a patient subgroup from the Ticlopidine Aspirin Stroke Study. Neurology. 1993;43(1 Part 1):27-. 10.1212/wnl.43.1_part_1.27 8423906

[26] Harbison JW. Ticlopidine versus aspirin for the prevention of recurrent stroke. Analysis of patients with minor stroke from the Ticlopidine Aspirin Stroke Study. Stroke. 1992;23(12):1723–7. 1448821 10.1161/01.str.23.12.1723

[27] Pan Y, Chen W, Xu Y, Yi X, Han Y, Yang Q, et al Genetic Polymorphisms and Clopidogrel Efficacy for Acute Ischemic Stroke or Transient Ischemic AttackClinical Perspective: A Systematic Review and Meta-Analysis. Circulation. 2017;135(1):21–33. 10.1161/CIRCULATIONAHA.116.024913 27806998

[28] Droy-Lefaix M. Effect of the antioxidant action of Ginkgo biloba extract (EGb 761) on aging and oxidative stress. Age. 1997;20(3):141–9. 10.1007/s11357-997-0013-1 23604306 PMC3455891

[29] Tang Y, Huang B, Sun L, Peng X, Chen X, Zou X. Ginkgolide B promotes proliferation and functional activities of bone marrow-derived endothelial progenitor cells: involvement of Akt/eNOS and MAPK/p38 signaling pathways. Eur Cell Mater. 2011;21:459–69. 21623570 10.22203/ecm.v021a34

[30] Rong Y, Geng Z, Lau BH. Ginkgo biloba attenuates oxidative stress in macrophages and endothelial cells. Free Radical Biology and Medicine. 1996;20(1):121–7. 8903688 10.1016/0891-5849(95)02016-0

[31] Schindowski K, Leutner S, Kressmann S, Eckert A, Müller WE. Age-related increase of oxidative stress-induced apoptosis in micePrevention by Ginkgo biloba extract (EGb761). Journal of neural transmission. 2001;108(8–9):969–78. 10.1007/s007020170016 11716149

[32] Huang C-H, Yang M-L, Tsai C-H, Li Y-C, Lin Y-J, Kuan Y-H. Ginkgo biloba leaves extract (EGb 761) attenuates lipopolysaccharide-induced acute lung injury via inhibition of oxidative stress and NF-κB-dependent matrix metalloproteinase-9 pathway. Phytomedicine. 2013;20(3–4):303–9. 10.1016/j.phymed.2012.11.004 23219342

[33] Brewer T, Colditz GA. Postmarketing surveillance and adverse drug reactions: current perspectives and future needs. Jama. 1999;281(9):824–9. 10071004 10.1001/jama.281.9.824

[34] An AR, Kim K, Lee J-H, Sung N-J, Lee S-i, Hyun MK. Having a usual source of care and its associated factors in Korean adults: a cross-sectional study of the 2012 Korea Health Panel Survey. BMC family practice. 2016;17(1):167 10.1186/s12875-016-0555-3 27899071 PMC5129206

