# Supplementary figures and images for: Incidence of neutropenia in patients with ticlopidine/Ginkgo biloba extract combination drug for vascular events: A post-marketing cohort study
Source: PLoS One. 2019 Jun 5;14(6):e0217723. doi: 10.1371/journal.pone.0217723 (PMC6550423; doi:10.1371/journal.pone.0217723)

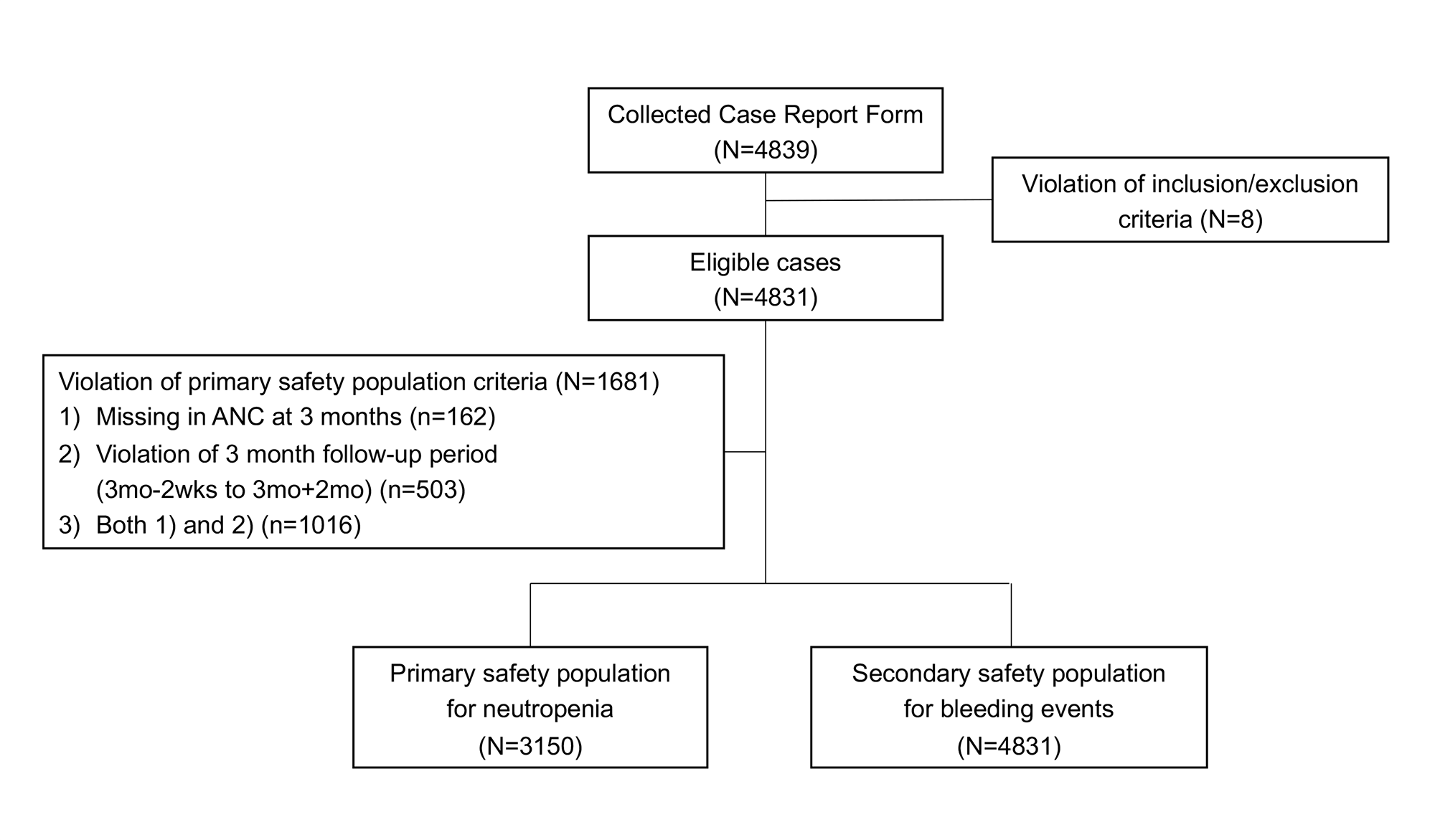

Supplement: S1 Fig — (TIF) [file pone.0217723.s001.tif]
