# Supplementary material for: Incidence of neutropenia in patients with ticlopidine/Ginkgo biloba extract combination drug for vascular events: A post-marketing cohort study
Source: PLoS One. 2019 Jun 5;14(6):e0217723. doi: 10.1371/journal.pone.0217723 (PMC6550423; doi:10.1371/journal.pone.0217723)
Supplement: S1 Table — (PDF) [file pone.0217723.s002.pdf]

S1 Table. Medication status of study drug

Medication status			
Continuation	4143 (85.6%)		
Premature discontinuation*	696 (14.4%)		
Adverse events		119 (17.1%)	
Neutropenia			8 (6.7%)
Bleeding events			11 (9.2%)
Others			100 (84.0%)
Vascular events		12 (1.7%)	
Stroke			8 (66.7%)
Myocardial infarction			0 (0%)
Cardiovascular death			0 (0%)
Others			4 (33.3%)
Follow-up loss		421 (60.5%)	
Refusal		39 (5.6%)	
Other specific causes		105 (15.1%)	

* Predefined in the case report form as 1) adverse events, 2) vascular events, 3) follow-up loss, 4) refusal or 5) other specific causes
