# Supplementary material for: Incidence of neutropenia in patients with ticlopidine/Ginkgo biloba extract combination drug for vascular events: A post-marketing cohort study
Source: PLoS One. 2019 Jun 5;14(6):e0217723. doi: 10.1371/journal.pone.0217723 (PMC6550423; doi:10.1371/journal.pone.0217723)
Supplement: S2 Table — (PDF) [file pone.0217723.s003.pdf]

S2 Table. Trial Drug Information

<Safety Population>

	Total (N=4831)
Yuclid (250mg/80mg Ticlopidine HCl/Ginkgo biloba ext.)	n (%)
Dosing frequency per day	
1	504 (10.5)
2	4292 (89.5)
Missing	35
Daily dose (tablet)	
0.5	1 (0.02)
1	1077 (22.5)
2	3717 (77.5)
3	1 (0.02)
Missing	35
Treatment duration	
≤5 days	13 (0.3)
≥6 days, <2 weeks	59 (1.5)
≥2 weeks, <30 days	115 (2.8)
≥30 days, <60 days	169 (4.2)
≥60 days, <90 days	963 (23.7)
≥90 days	2739 (67.5)
Missing	773
