# Supplementary material for: Incidence of neutropenia in patients with ticlopidine/Ginkgo biloba extract combination drug for vascular events: A post-marketing cohort study
Source: PLoS One. 2019 Jun 5;14(6):e0217723. doi: 10.1371/journal.pone.0217723 (PMC6550423; doi:10.1371/journal.pone.0217723)
Supplement: S3 Table — (PDF) [file pone.0217723.s004.pdf]

S3 Table. Details of subject information with neutropenia at 3 months

Sex	Age	Weight	Care setting	Indication	Drug initiation	Dose	Follow-up	ANC at 3 months (mm^3/L)
Female	60	53kg	OPD	TIA/Stroke	2009/11/23	500mg/d	2010/02/22	596
Male	43	107kg	Inpatient	CHD	2009/10/28	500mg/d	2010/01/25	1168
Male	71	52kg	Inpatient	TIA/Stroke	2009/12/21	500mg/d	2010/04/01	548
Male	56	58kg	OPD	TIA/Stroke	2009/08/07	500mg/d	2009/11/10	1084
Female	66	NA	OPD	TIA/Stroke	2009/12/30	500mg/d	2010/04/07	581
Male	68	68kg	OPD	CHD	2010/11/08	500mg/d	2011/01/31	1195
Female	80	51kg	Both	CHD	2011/12/27	500mg/d	2012/03/20	1170
Male	61	65kg	OPD	TIA/Stroke	2010/07/09	500mg/d	2010/10/13	614
Male	63	63kg	Inpatient	TIA/Stroke	2014/10/29	500mg/d	2015/01/22	982

ANC=Absolute Neutrophil Count, OPD=Outpatient Department, TIA=Transient Ischemic Attack, CHD=Coronary Heart Disease.
