# Supplementary material for: Incidence of neutropenia in patients with ticlopidine/Ginkgo biloba extract combination drug for vascular events: A post-marketing cohort study
Source: PLoS One. 2019 Jun 5;14(6):e0217723. doi: 10.1371/journal.pone.0217723 (PMC6550423; doi:10.1371/journal.pone.0217723)
Supplement: S4 Table — (PDF) [file pone.0217723.s005.pdf]

S4 Table. Bleeding events by sex, age group and treatment duration

<Secondary safety population>

Subgroups	Cases	Bleeding events, N (%) [95% CI of %]				P-value
		Any bleeding	Severe[*]	Moderate[*]	Mild[*]	
Sex						0.045[†]
Male	2513	8 (0.32) [0.14, 0.63]	2 (25)	2 (25)	4 (50)	0.61[‡]
Female	2318	17 (0.73) [0.43, 1.17]	1 (5.9)	1 (5.9)	15 (88.2)	<0.01[‡]
Age group						0.88[†]
40 – 49	304	2 (0.66) [0.08, 2.36]	0 (0)	1 (50)	1 (50)	1.00[‡]
50 – 59	910	5 (0.55) [0.18, 1.28]	0 (0)	0 (0)	5 (100)	-
60 – 69	1410	6 (0.43) [0.16, 0.92]	1 (16.7)	1(16.7)	4 (66.7)	0.22[‡]
70 – 79	1704	8 (0.47) [0.20, 0.92]	1 (12.5)	1(12.5)	6 (75)	0.04[‡]
80 or more	503	4 (0.80) [0.22, 2.02]	1 (25)	0 (0)	3 (75)	0.32[‡]
Duration						<0.01[†]
≤ 5 days	13	0 (0)	0 (0)	0 (0)	0 (0)	-
≥ 6 days, < 2 weeks	59	0 (0)	0 (0)	0 (0)	0 (0)	-
≥ 2 weeks, < 30 days	115	1 (0.87) [0.02, 4.75]	0 (0)	0 (0)	1 (100)	-
≥ 30 days, < 60 days	169	6 (3.55) [1.31, 7.57]	1 (16.7)	1 (16.7)	4 (66.7)	0.22[‡]
≥ 60 days, < 90 days	963	5 (0.52) [0.17, 1.21]	1 (20)	0 (0)	4 (80)	0.18[‡]
≥ 90 days	2739	10 (0.37) [0.18, 0.67]	1 (10)	1 (10)	8 (80)	0.01[‡]
Missing	773	3	-	-	-	-

[*]Note: Denominator of percentage is the number of subjects who had bleeding event.

† For difference by subgroup

‡ For difference by bleeding severity
