# Supplementary material for: Incidence of neutropenia in patients with ticlopidine/Ginkgo biloba extract combination drug for vascular events: A post-marketing cohort study
Source: PLoS One. 2019 Jun 5;14(6):e0217723. doi: 10.1371/journal.pone.0217723 (PMC6550423; doi:10.1371/journal.pone.0217723)
Supplement: S5 Table — (PDF) [file pone.0217723.s006.pdf]

S5 Table. Characteristics of the included studies for meta-analysis

**Hass 1989**	
Methods	Randomized, double blinded trial
Participants and study outcomes	3069 patients (1529 in Ticlopidine, 1540 in Aspirin)<br>1982 – 1986<br>Indication: Recent transient ischemia or mild persistent focal cerebral or retinal ischemia<br>Ticlopidine group:<br>- Mean age: 63 yrs<br>- Male: 64%<br>- Race: 80% white<br>Study outcomes<br>- Primary: non-fatal stroke or death from all causes<br>- Secondary: fatal and non-fatal stroke and MI
Ticlopidine Rx	Ticlopidine 250mg twice per day<br>Duration of Rx: mean 29 months
Neutropenia	Mild-to-moderate neutropenia (absolute neutrophil count, 450 to 1200 per mm^3)<br>- 22 in ticlopidine group<br>Severe neutropenia (absolute neutrophil count, <450 per mm^3)<br>- 13 in ticlopidine group (8 women and 5 men)<br>- Mean age 65 yrs (range, 45 – 81)<br>- All episodes occurred between 1 and 3 months after ticlopidine use and resolved within 3 weeks after discontinuation
Notes	Complete blood counts were done locally every two weeks for the first 3 months
**Gent 1989**	
Methods	Randomized, double blinded trial
Participants and study outcomes	1053 patients (525 in Ticlopidine, 1540 in Placebo)<br>Indication: Recent thromboembolic stroke<br>Ticlopidine group:<br>- Mean age: 66 yrs<br>- Male: 60%<br>- Race: 73% Caucasian<br>Study outcomes<br>- Primary: occurrence of stroke, myocardial infarction, or vascular death<br>- Secondary: outcomes of stroke, myocardial infarction, or death from any cause; stroke or stroke death; vascular death; and death from any cause

Ticlopidine Rx	Ticlopidine 250mg twice per day<br>Duration of Rx on study drug: mean 17 months (follow-up 24 months)<br>Compliance: 88% (by pill counts when on study drug)<br>Early permanent discontinuation of study drug: 52%
Neutropenia	Moderate neutropenia (absolute neutrophil count, 450 to 800 per mm^3)<br>- 1 in ticlopidine group<br>Severe neutropenia (absolute neutrophil count, <450 per mm^3)<br>- 4 in ticlopidine group<br>- All episodes resolved within days after discontinuation
Notes	Laboratory assessments: every 2 weeks for the first 3 months, at months 4,5,6, and 8, and every 4 months thereafter.
**Gorelick 2003**	
Methods	Randomized, double blinded trial
Participants and study outcomes	1809 patients (902 in Ticlopidine, 907 in Placebo)<br>1995 – 2001<br>Indication: Recent noncardioembolic stroke<br>Ticlopidine group:<br>- Mean age: 60.9 yrs<br>- Male: 55.5%<br>- Race: All African American<br>Study outcomes<br>- Primary: composite outcome of recurrent stroke, myocardial infarction, or vascular death<br>- Secondary: recurrent stroke or death; non-fatal or fatal stroke; recurrent stroke, myocardial infarction, or death from all causes; vascular death; death from all causes; or myocardial infarction
Ticlopidine Rx	Ticlopidine 250mg twice per day<br>Duration of Rx: 41% of ticlopidine group completed 24 months follow-up (median 710 days)<br>Compliance: 90% (by pill counts)
Neutropenia	Serious neutropenia (absolute neutrophil count, <1000 per mm^3)<br>- 31 in ticlopidine group<br>All episodes were reversible
Notes	Complete blood count: every 2 weeks during the first 3 months of the study or at any unscheduled time as needed
**Fukuuchi 2008**	
Methods	Randomized, double blind trial
Participants and study outcomes	1151 patients (578 in Ticlopidine, 573 in Clopidogrel)<br>Indication: non-cardioembolic stroke<br>Ticlopidine group:<br>- Mean age: 65 yrs<br>- Male: 74%

	- Race: All Japanese<br>Study outcomes<br>- Primary safety: the safety of each medication at 52 weeks, with emphasis on hematologic changes, hepatic dysfunction, nontraumatic hemorrhage (leading to or prolonging hospitalization or resulting in death), and other serious adverse drug reactions (i.e., considered to be life-threatening, leading to or pro- longing hospitalization, or resulting in irreversible impairment or death)
Ticlopidine Rx	Ticlopidine 200mg once daily<br>Duration of Rx: 52 weeks<br>Premature discontinuation: 233 (40.3%)
Neutropenia	Neutropenia (absolute neutrophil count, <1500 per mm^3)<br>- 14 in ticlopidine group (2.4%)<br>Severe neutropenia (absolute neutrophil count, <500 per mm^3)<br>- 0 in ticlopidine group
Notes	Hematologic tests were scheduled every 2 weeks for the first 8 weeks of treatment and subsequently every 12 weeks from week 12.
