# Supplementary material for: Incidence of neutropenia in patients with ticlopidine/Ginkgo biloba extract combination drug for vascular events: A post-marketing cohort study
Source: PLoS One. 2019 Jun 5;14(6):e0217723. doi: 10.1371/journal.pone.0217723 (PMC6550423; doi:10.1371/journal.pone.0217723)
Supplement: S6 Table — (PDF) [file pone.0217723.s007.pdf]

S6 Table. Neutropenia incidence defined with different cut-offs of absolute neutrophil count and comparison to the previous studies.

Study	ANC cut-off	Reported neutropenia incidence of ticlopidine (% [95% CI])	Neutropenia incidence of ticlopidine/Ginkgo biloba using the same cut-offs (% [95% CI])
**Hass 1989 (TASS)**	1200/mm^3	2.29 [1.59 – 3.18]	0.29 [0.13 – 0.54] (main-result)
**Gent 1989 (CATS)**	800/mm^3	0.76 [0.21 – 1.95]	0.13 [0.03 – 0.33]
**Gorelick 2003**	1000/mm^3	3.44 [2.34 – 4.88]	0.16 [0.05 – 0.37]
**Fukuuchi 2008**	1500/mm^3	2.18 [1.41 – 3.35]	1.08 [0.75 – 1.51]
